# Rare Association of two Genetic Causes of Sudden Death in a Young Survivor

**DOI:** 10.5935/abc.20170016

**Published:** 2017-02

**Authors:** Dulce Brito, Andreia Magalhães, Nuno Cortez-Dias, Gabriel Miltenberger-Miltenyi

**Affiliations:** 1 Cardiology Department - Hospital Universitário de Santa Maria, Cardiovascular Center of Lisbon University (CCUL), Portugal; 2 Instituto de Medicina Molecular (IMM) - Faculdade de Medicina de Lisboa, Portugal

**Keywords:** Death, Sudden Cardiac, Cardiomyopathy, Hypertrophic, Familial, Adolescent, Brugada Syndrome

## Introduction

Sudden cardiac arrest (SCA) in young adults is frequently caused by inherited cardiac diseases, particularly cardiomyopathies and ion channelopathies.^1^ Genetic testing can be essential in the follow-up of survivors and today´s genetic diagnostics may include the parallel analysis of several SCA related genes, most commonly those associated with ion channelopathies and hypertrophic cardiomyopathy (HC). We present the case of a young survivor of SCA, carrier of double heterozygosity for mutations in the *SNC5A* and *MYBPC3* genes, illustrating the complexity of genotype-phenotype associations and the difficulties of decisions regarding therapeutic interventions in inherited cardiac diseases.

## Case Report

**Figure 1:**
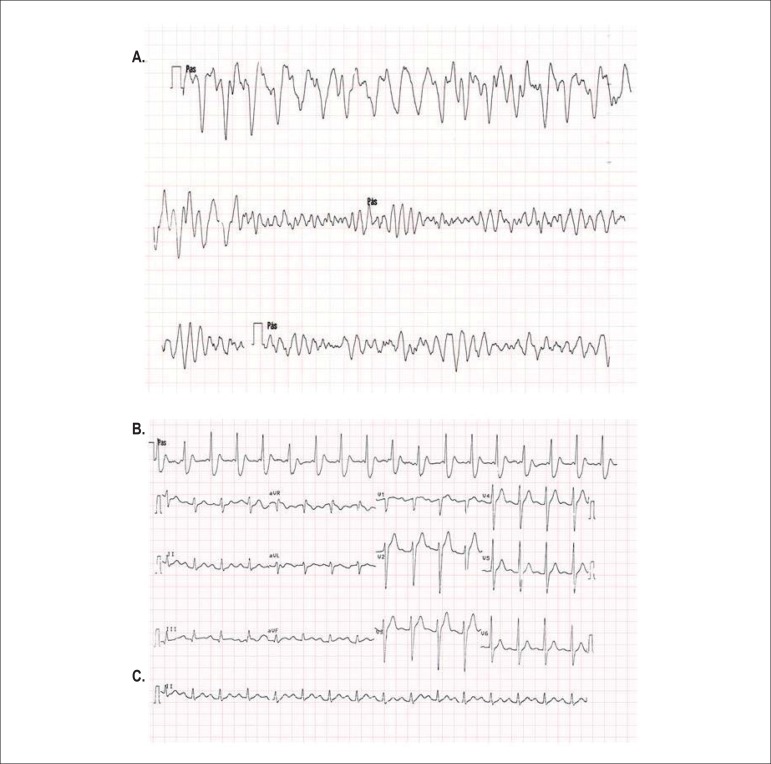
A: Polymorphic ventricular tachycardia degenerating into ventricular fibrillation (rhythm strips in sequence); B: Rhythm strip after defibrillation; C: ECG at hospital admission (PR interval: 0.26-0.28 sec; QTc: 0.45 sec).

A healthy 19-year-old man suffered SCA while playing football. His girlfriend (a medical student) carried out cardiopulmonary resuscitation until the arrival of the ambulance. Polymorphic ventricular tachycardia degenerating into ventricular fibrillation (VF) was documented (Figure 1A) and defibrillation was successfully performed (Figure 1B). At subsequent hospital admission, the electrocardiogram (ECG) showed sinus rhythm (95 bpm), with a PR interval of 0.26-0.28 sec and a QTc interval of 0.45 sec. (Figure 1C). The echocardiographic study (echo) was normal and reversible causes of SCA including ionic, infectious and toxic were excluded. The patient had a normal clinical exam and no personal history of severe illness. He took no medication. There was no family history of cardiac disease or sudden death. Thoracic X-ray, cardiac magnetic resonance, exercise test (treadmill) and coronary angiography were normal.

Electrophysiological study (EPS) showed a prolonged HV interval (80 ms) and ventricular stimulation (600 ms cycle - 250-220-220 at RV apex) induced polymorphic VT with no pulse (successfully terminated by external cardioversion). A provocative test for Brugada syndrome (BrS) was postponed.

**Figure 2:**
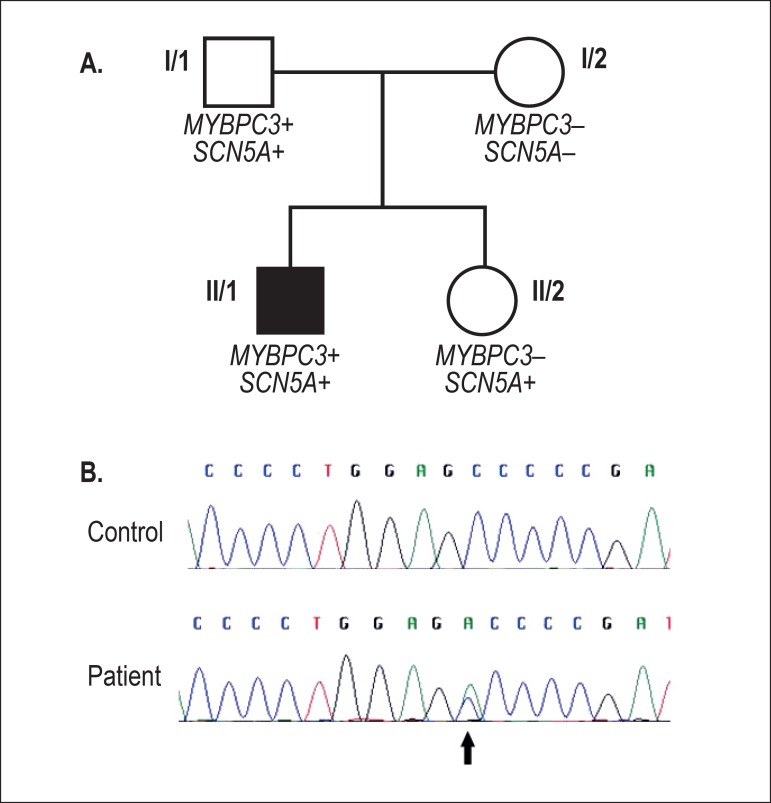
A: Pedigree of the family. MYBPC3+: harboring the MYBPC3 mutation, SCN5A+: harboring the SCN5A mutation; B: the MYBPC3 mutation p.Ala149Asp. Upper lane: healthy individual; lower lane: index patient.

After written informed consent, genetic screening was performed on a panel of 9 genes: *MYBPC3, MYH7, MYL2, SCN5A, TNNI3, TNNT2, KCNQ1, KCNH2* and *LQT5*. The entire coding regions were tested by PCR and direct sequencing. We found the mutation c.3622G>T; p.Glu1208* in the *SCN5A* gene (NM_198056.2), that was already described in BrS. Additionally, another sequent variant, c.446C>A; p.Ala149Asp, in the *MYBPC3* gene (NM_000256.3) was detected (Figure 2B). This alteration was reported as a rare sarcomeric gene variant in a single case of the offspring cohort of the Framingham Heart Study, including 1,637 unrelated individuals.^2^ Although this single individual did not show alteration of the left ventricle wall thickness, only one wall segment was measured, without any further detailed information, thus HC cannot be excluded. Besides, no information was given whether the index case of this family was also harboring this sequence variant in *MYBPC3*.

The p.Ala149Asp mutation in *MYBPC3* affects an evolutionarily conserved amino acid and it was absent in 100 age-matched Portuguese control samples. Regarding the various in-silico mutation prediction programs, PolyPhen2 (http://genetics.bwh.harvard.edu/pph2/) predicted this alteration as possibly damaging with a score of 0.65 (sensitivity: 0.87, specificity: 0.91). The SNPs&Go program (http://snps.biofold.org/snps-and-go//snps-and-go.html) predicted this variant as disease associated variation (0.696).

Subsequent family analysis showed that the patient´s father was carrier of both *SCN5A* and *MYBPC3* mutations (Figure 2A). The father was submitted to EPS with provocative test with flecainide. This test showed negative results. The sister of our index patient was harboring the *SCN5A* mutation solely. Because of the young age, we decided not to perform provocative tests or EPS.

A cardioverter defibrillator (ICD) was implanted in the index patient. Provocative pharmacological tests were systematically refused by the patient thereafter. Serial ECGs during hospitalization showed normal patterns except for mild PR prolongation that persisted also during ambulatory follow up. Family screening (parents and sister) revealed normal clinics, ECG and echo studies.

## Discussion

In the patient presented herein, the persistent prolonged PR interval on serial ECGs that is explained by a prolonged H-V interval during EPS, the induction of sustained polymorphic VT during EPS (that had to be terminated by an external DC shock) and the identification of a pathogenic non-sense mutation in *SCN5A* gene (already described as causing an important reduction in sodium currents - INa)^3^ favored the diagnosis of a loss-of-function sodium channelopathy like BrS or progressive cardiac conduction disease. These inherited conditions may overlap and can coexist in the same family and even in the same individual and it was suggested that they may indeed represent different aspects of the same disease and not separate entities. However, with no BrS sign on ECG and as the patient refused a provocative test, a clear diagnosis of BrS was not confirmed.^4^

Disease penetrance and expressivity are highly variable in these diseases and the causal role of *SCN5A* mutations in BrS is not yet clearly established. The patient´s father, although with the same mutation, was healthy and a provocative test for BrS was negative. The young sister of the patient had also a normal phenotype although invasive tests were not performed. Additionally, both the patient and the father are carriers of a missense mutation in the *MYBPC3* gene, one common mutated gene in HC, an autosomal-dominant inherited disease that may cause ventricular arrhythmias and SCA mainly in the young. The identified mutation supported the probability of pathogenicity. In HC carriers of a mutant gene, the phenotype may develop late in life particularly with *MYBPC3* gene mutations. However, under the "appropriate" trigger, like strenuous exercise as was the case with our patient, sudden death may be the first manifestation of the disease. To our knowledge, this is the first report on *SCN5A* and *MYBPC3* double mutations.

Genetic tools and protocols are evolving fast.^5^ The new era of genetic testing, with the easy possibility of screening a large number of genes, like next generation sequencing, is greatly enhancing the perspectives of a genetic diagnosis in inherited cardiomyopathies in a fast and cost-efficient way. However, it is also increasing the complexity of interpretation namely in the context of a limited or even absent phenotype, thus caution should be kept when considering clinical decisions.
